# ALKBH1-8 and FTO: Potential Therapeutic Targets and Prognostic Biomarkers in Lung Adenocarcinoma Pathogenesis

**DOI:** 10.3389/fcell.2021.633927

**Published:** 2021-06-03

**Authors:** Geting Wu, Yuanliang Yan, Yuan Cai, Bi Peng, Juanni Li, Jinzhou Huang, Zhijie Xu, Jianhua Zhou

**Affiliations:** ^1^Department of Pathology, Xiangya Hospital, Central South University, Changsha, China; ^2^Department of Pharmacy, Xiangya Hospital, Central South University, Changsha, China; ^3^Department of Oncology, Mayo Clinic, Rochester, MN, United States; ^4^National Clinical Research Center for Geriatric Disorders, Xiangya Hospital, Central South University, Changsha, China

**Keywords:** AlkB family, lung adenocarcinoma, expression profiles, prognosis, methylation, immune cell infiltration

## Abstract

The AlkB family consists of Fe(II)- and α-ketoglutarate-dependent dioxygenases that can catalyze demethylation on a variety of substrates, such as RNA and DNA, subsequently affecting tumor progression and prognosis. However, their detailed functional roles in lung adenocarcinoma (LUAD) have not been clarified in a comprehensive manner. In this study, several bioinformatics databases, such as ONCOMINE, TIMER, and DiseaseMeth, were used to evaluate the expression profiles and prognostic significance of the AlkB family (ALKBH1-8 and FTO) in LUAD. The expression levels of ALKBH1/2/4/5/7/8 were significantly increased in LUAD tissues, while the expression levels of ALKBH3/6 and FTO were decreased. The main functions of differentially expressed AlkB homologs are related to the hematopoietic system and cell adhesion molecules. We also found that the expression profiles of the AlkB family are highly correlated with infiltrating immune cells (i.e., B cells, CD8 + T cells, CD4 + T cells, macrophages, neutrophils and dendritic cells). In addition, DNA methylation analysis indicated that the global methylation levels of ALKBH1/2/4/5/6/8 and FTO were decreased, while the global methylation levels of ALKBH3/7 were increased. In addition, the patients with upregulated ALKBH2 have significantly poor overall survival (OS) and post-progressive survival (PPS). Taken together, our work could provide insightful information about aberrant AlkB family members as potential biomarkers for the diagnostic and prognostic evaluation of LUAD. Especially, ALKBH2 could be served as a therapeutic candidate for treating LUAD.

## Introduction

Lung cancer is one of the most common malignant tumors in the world (Yan et al., 2019). Thousands of patients die from this malignant disease every year. In addition, the incidence of lung cancer continues to increase (Lin et al., 2019; Subedi et al., 2019; Arbyn et al., 2020). Lung adenocarcinoma (LUAD) is the most common histological subtype of lung cancer (Wei et al., 2019). At present, great progress has been made in the diagnosis and treatment of LUAD (Lim and Ma, 2019; Zhang et al., 2019b), but there is still a lack of advanced diagnosis and treatment programs (Oberndorfer and Mullauer, 2018). Therefore, it is urgent to identify more therapeutic targets and prognostic biomarkers.

The AlkB family of Fe(II) and α-ketoglutarate-dependent dioxygenases is a universal class of direct reversal DNA repair enzymes (Pilzys et al., 2019; Xu et al., 2020). This family of enzymes can remove alkyl adducts from nucleobases by oxidative dealkylation, protecting the bacterial genome from alkylation damage (Trewick et al., 2002; Liu et al., 2018). Research has found that there are currently 9 homologs of the AlkB protein, including ALKBH1-8 and FTO (Du et al., 2019; Xiao et al., 2020). They have multiple biological functions, such as regulation of RNA metabolism, involvement in the DNA damage response or participation in fatty acid metabolism (Wu et al., 2016; Bian et al., 2019; Rajecka et al., 2019).

Many studies have found that the AlkB family plays a key role in the occurrence and development of tumors either directly or indirectly, including breast cancer, ovarian cancer, and bladder cancer (Fujii et al., 2013; Tao et al., 2016; Zhu et al., 2019). However, the detailed role of the AlkB family in LUAD remains to be further elucidated. With the rapid development of second-generation gene sequencing technology and the establishment of various databases, a comprehensive analysis of the AlkB family is beneficial to the clarification of the AlkB family in LUAD pathogenesis and treatment. In this study, we conducted a thorough and comprehensive bioinformatics analysis of the AlkB family (including ALKBH1-8 and FTO) in LUAD. Moreover, we evaluated their potential as therapeutic targets and prognostic biomarkers based on multiple public bioinformatics databases. The purpose of this study is to help clinicians choose appropriate therapeutic drugs and more accurately predict the long-term prognosis of patients with LUAD.

## Materials and Methods

### Oncomine3.0

Oncomine3.0^1^ is a cancer microarray database and web-based data mining platform with 40 microarray data sets and approximately 100 differential expression analyses. It provides users with powerful, comprehensive genome-wide expression analysis (Rhodes et al., 2004, 2007). In this research, *p* < 0.05 and genes ranked in the top 10% were taken as the significance thresholds. Student’s *t* test was used to analyze the difference in AlkB family expression in LUAD. Specific information is summarized in Supplementary Table 1.

### GEPIA2

GEPIA2^2^, Gene Expression Profiling Interactive Analysis, is a web-based that provides key interactive and customizable features, including differential expression analysis, correlation analysis, and patient survival analysis (Tang et al., 2017, 2019). In this study, we used the “single gene analysis” module of GEPIA to analyze mRNA expression differences between tumors and normal tissues. At the same time, multiple gene comparison analysis of the AlkB family was performed using the “Multiple Gene Comparison” module and the “KIRC” GEPIA dataset. Student’s *t* test was used to generate *p* values for expression, and *p* < 0.05 was considered statistically significant.

### UALCAN

UALCAN^3^ is an interactive network resource based on TCGA datasets that can compare the primary tumor and normal tissue samples based on pathological stage, tumor grade and other clinicopathological characteristics (Chandrashekar et al., 2017). In our research, the expression data of the AlkB family were obtained through the “stage analysis” module and the “KIRC” data set of UALCAN. Differences in transcriptional expression were compared by Student’s *t* test, and *p* < 0.05 was considered statistically significant.

### The Human Protein Atlas

The Human Protein Atlas is a database of tools that can be used to identify clinically useful biomarkers using produced antibody and protein expression data (Asplund et al., 2012). Researchers can study the expression patterns of different proteins expressed in specific tumors. In this study, we directly compared the protein expression of Alkb family members in normal and LUAD tissues by immunohistochemistry.

### Kaplan-Meier Plotter

Kaplan-Meier plotter^4^ was used to analyze the prognostic value of the AlkB family in LUAD (Sun et al., 2019; Wang et al., 2019). To analyze OS and PPS in patients with LUAD, the patient samples were divided into two groups by median expression (high and low expression) and were evaluated by the K-M survival chart. Information on the number of high-risk cases, median mRNA expression levels, HR, 95% CI, and *p* values can be found on the K-M plotter website. A *p* < 0.05 was considered statistically significant.

### cBioPortal

c-BioPortal^5^, a comprehensive network of resources, can be used to explore, visualize and analyze multidimensional cancer genomics and clinical data. It contains more than 200 cancer genomics studies, including all the data on TCGA (Cerami et al., 2012; Wu et al., 2019). In this study, we analyzed the genome map of the AlkB family, which includes mutation and mRNA expression data. The mRNA expression z scores (RNA Seq V2 RSEM) were obtained using a z score threshold of ± 0.75.

### GeneMANIA

Given a list of genes to query, GeneMANIA^6^ can use vast amounts of genomics and proteomics data to find genes with similar functions. In addition, GeneMANIA can predict gene function. Given a query gene, GeneMANIA finds genes that might share the target gene’s function based on their interactions (Zuberi et al., 2013; Franz et al., 2018).

### Cytoscape

Cytoscape can integrate biomolecular interaction networks with high-throughput expression data and other molecular states into a unified conceptual framework (Shannon et al., 2003). In this study, we performed functional integration on 284 co-expressed molecules of AlkB family members screened from cBioPortal (the molecular names are provided in Supplementary Table 2). According to the degree values between the interacting proteins decided the nodes size. The higher the degree, the larger the circles.

### WebGestalt

WebGestalt^7^ is a comprehensive, powerful, flexible, and interactive web-based analysis toolkit (Wang et al., 2017). Gene ontology (GO) enrichment analysis and Kyoto Encyclopedia of Genes and Genomes (KEGG) pathway analysis were performed in this study using WebGestalt.

### TIMER2.0

TIMER2.0^8^ can assess immune cell infiltration and the clinical impact of 10,897 tumors from 32 cancer types (Li et al., 2017). In our study, the “Gene Module” was used to assess the association between the AlkB family and immune cell infiltration. The “survival module” was used to assess the correlation of clinical outcomes with immune cell infiltration and the AlkB family. The multivariable cox proportional hazard model is used as the statistical method.

### DiseaseMeth2.0

DiseaseMeth2.0^9^ aims to provide information on abnormal DNA methylation in human diseases, especially all sorts of cancer, in the most complete collection and comments (Lv et al., 2012; Xiong et al., 2017). We used Wanderer to screen the possible methylation value of the AlkB family. A *p* < 0.05 was considered as statically significant.

## Results

### Abnormal Expression of the AlkB Family in Patients With LUAD

**FIGURE 1:**
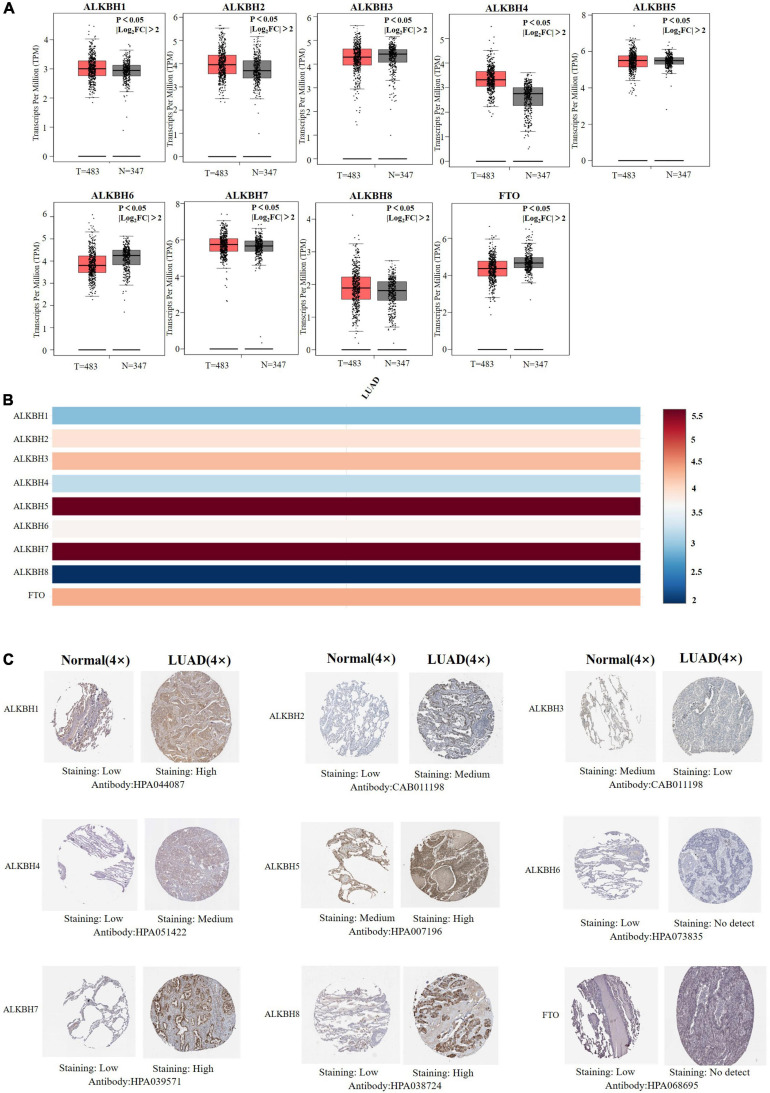
**(A)** mRNA expression levels of different AlkB family members in LUAD and normal lung samples (GEPIA). T and N indicated the LUAD tissues and normal tissues, respectively. **(B)** The relative expression of the AlkB family in LUAD. **(C)** Immunohistochemical expression of ALKB family in LUAD tissue and normal lung tissue (the Human Protein Atlas).

**FIGURE 2:**
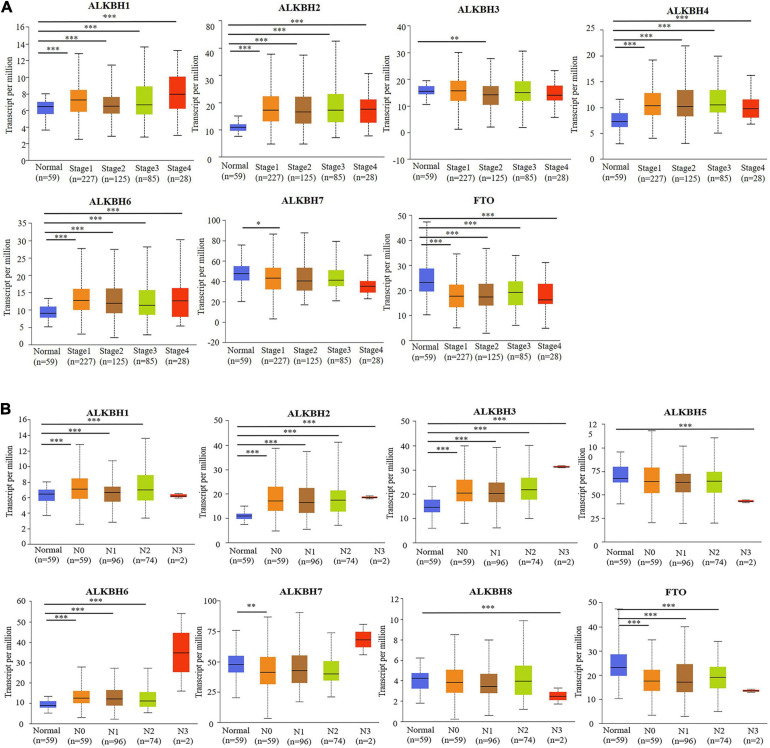
**(A)** The relationship between mRNA expression and pathological stage of LUAD patients with different members of the AlkB family (UALCAN). **(B)** The relationship between mRNA expression of distinct AlkB family members with lymph node metastasis of LUAD patients (UALCAN). ^∗^*p* < 0.05, ^∗∗^*p* < 0.01, ^∗∗∗^*p* < 0.001.

We first searched the expression levels of the AlkB family (ALKBH1-ALKBH8, FTO) in LUAD and normal lung tissue using the Oncomine database. The results are shown in Table 1. The results demonstrated that the expression levels of ALKBH1/2/4/5/7/8 were significantly elevated in LUAD. Five datasets showed that ALKBH1 expression in LUAD was higher than that in normal lung tissue (Garber et al., 2001; Su et al., 2007; Landi et al., 2008; Hou et al., 2010; Okayama et al., 2012). In the Selamat and Hou datasets, the expression level of ALKBH2 in LUAD was significantly upregulated (Hou et al., 2010; Selamat et al., 2012). The results of Selamat (Selamat et al., 2012), Okayama (Okayama et al., 2012), Hou (Hou et al., 2010), and Su (Su et al., 2007) all suggest that ALKBH4 expression was significantly higher in LUAD compared with normal samples. The expression levels of ALKBH5 and ALKBH7 in LUAD were markedly higher than those in normal lung tissues in the Okayama (Okayama et al., 2012) and Hou (Hou et al., 2010) datasets. However, Selamat et al. (2012) found a decreased level of ALKBH8 in LUAD. Next, mRNA expression levels of the AlkB family in LUAD and normal lung tissue were verified in the GEPIA database. As shown in Figure 1A, the results showed clear support that the expression levels of ALKBH1/2/4/5/7/8 in LUAD were significantly elevated, while the expression levels of ALKBH3/6 and FTO were reduced compared with normal lung tissues. GEPIA was also used to compare the relative expression levels of the AlkB family in LUAD, and this analysis revealed that ALKBH5/7 had the highest relative expression levels among all AlkB family molecules (Figure 1B). After a comprehensive analysis of the mRNA expression levels of Alkb family in LUAD, we used the Human Protein Atlas to explore the protein expression levels of Alkb family in LUAD. The results showed that, ALKB1/2/4/5/7/8 was medium or high expression in LUAD. However, ALKB3/6 and FTO had low or no detect expression in LUAD. The results are shown in Figure 1C. This result is consistent with our previous findings on mRNA levels of expression. Moreover, UALCAN was used to analyze the relationship between the mRNA expression of AlkB family members and the clinicopathological staging of LUAD. From the results, it is clear that the mRNA expression level of ALKBH1/2/4/6 was positively correlated with tumor stage. In contrast, the mRNA expression levels of ALKBH7 and FTO were negatively correlated with tumor stage (Figure 2A). Similarly, the mRNA expression level of AlkB family members was also significantly correlated with lymph node metastasis. The highest mRNA expression levels of ALKBH2 and ALKBH3 were found in tumor N3 and were significantly correlated. At the same time, the mRNA expression levels of ALKBH2, ALKBH3, and ALKBH6 had a trend to upper expression in tumors with lymph node metastasis, although this was not significant. That may be caused by insufficient sample size and other reasons. Conversely, the expression levels of ALKBH5, ALKBH8 and FTO were lower than those in normal tissues at any stage of lymph node metastasis (Figure 2B).

**TABLE 1:** The expression profiles of the AlkB family in patients with LUAD using the Oncomine database.

**Name**	**Dataset**	**Fold change**	***p* values**	**References**
ALKBH1	Garber Lung	4.353	1.61E-4	Garber et al. (2001)
	Okayama Lung	1.368	5.58E-8	Okayama et al. (2012)
	Su Lung	1.100	0.035	Su et al. (2007)
	Landi Lung	1.069	0.016	Landi et al. (2008)
	Hou Lung	1.108	0.009	Hou et al. (2010)
ALKBH2	Selamat Lung	1.204	6.56E-5	Selamat et al. (2012)
	Hou Lung	1.211	9.00E-4	Hou et al. (2010)
ALKBH4	Su Lung	1.684	8.00E-4	Su et al. (2007)
	Hou Lung	1.181	8.88E-7	Hou et al. (2010)
	Selamat Lung	1.113	7.63E-4	Selamat et al. (2012)
	Okayama Lung	1.226	0.002	Okayama et al. (2012)
ALKBH5	Okayama Lung	1.227	0.004	Okayama et al. (2012)
	Hou Lung	1.093	0.006	Hou et al. (2010)
ALKBH7	Okayama Lung	1.117	0.015	Okayama et al. (2012)
	Hou Lung	1.052	0.047	Hou et al. (2010)
ALKBH8	Selamat Lung	1.219	1.19E-5	Selamat et al. (2012)

### Prognostic Value of mRNA Expression of the AlkB Family in LUAD Patients

**FIGURE 3:**
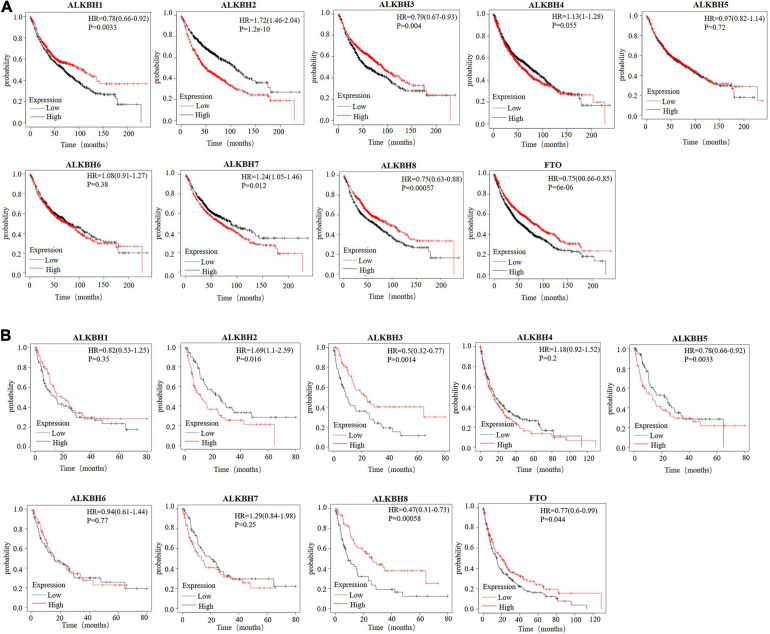
**(A)** The overall survival curve of AlkB molecules in LUAD patients (Kaplan-Meier plotter). **(B)** The post-progressive survival curve of the AlkB molecules in LUAD patients (Kaplan-Meier plotter).

Next, we used Kaplan-Meier plotter to analyze the prognostic values of the mRNA expression of AlkB family members in LUAD patients, including overall survival (OS) and post-progressive survival (PPS). The curves of OS are presented in Figure 3A. It is interesting to note that patients with higher transcription levels of ALKBH2, ALKBH4, and ALKBH7 displayed shorter OS times. The downregulations of ALKBH1, ALKBH3, ALKBH8, and FTO were significantly correlated with longer OS. However, ALKBH5 and ALKBH6 mRNA transcription showed no obvious correlation with the OS of LUAD patients. Moreover, the higher the transcription level of ALKBH2, the shorter the PPS in patients, while the higher the transcription levels of ALKBH3, ALKBH8 and FTO, the longer the PPS in patients. These results are summarized in Figure 3B.

### Genetic Alteration and Functional Analysis of the AlkB Family in LUAD Patients

**FIGURE 4:**
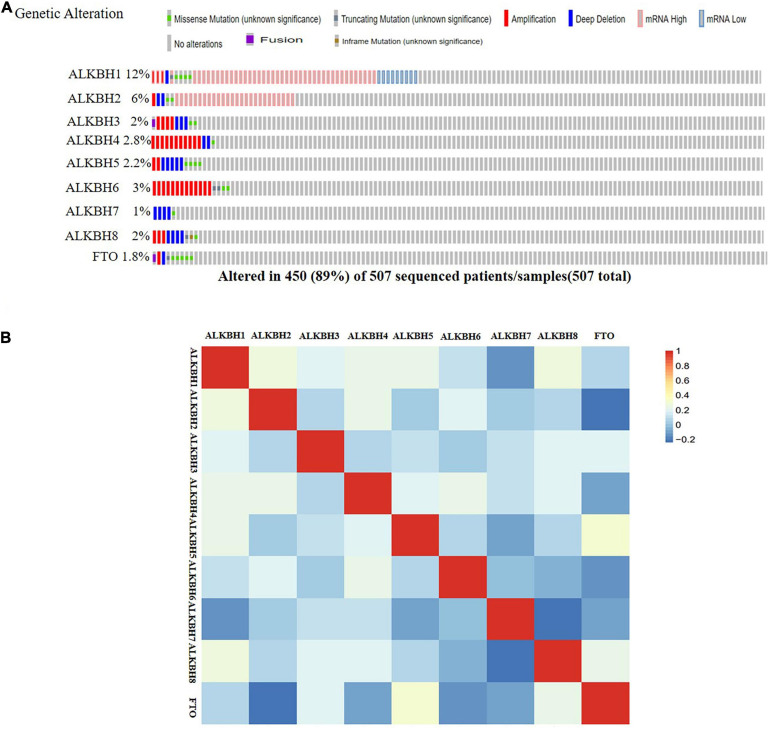
Genetic alternatives and correlation analysis of the AlkB family in LUAD. **(A)** Summary of the mutation rates in each AlkB family member in LUAD. **(B)** Correlation between different AlkB family in LUAD (GEPIA).

We conducted a comprehensive biological function analysis to further grasp the molecular characteristics of the differentially expressed AlkB family. The genetic alterations of the differentially expressed AlkB family were evaluated in the temporary TCGA dataset. The results are shown in Figure 4A. ALKBH1, ALKBH2, ALKBH3, ALKBH4, ALKBH5, ALKBH6, ALKBH7, ALKBH8, and FTO were all altered, with 12, 6, 2, 2.8, 2.2, 3, 1, 2, and 1.8% alterations in the LUAD samples, respectively. We calculated the correlation by mRNA expression in the GEPIA online database, and Pearson’s correction was included. There was a strongly negative correlation between ALKBH2 and FTO, ALKBH7 and ALKBH8. ALKBH5 was positively correlated with FTO, and ALKBH1 was positively correlated with ALKBH8 (Figure 4B).

**FIGURE 5:**
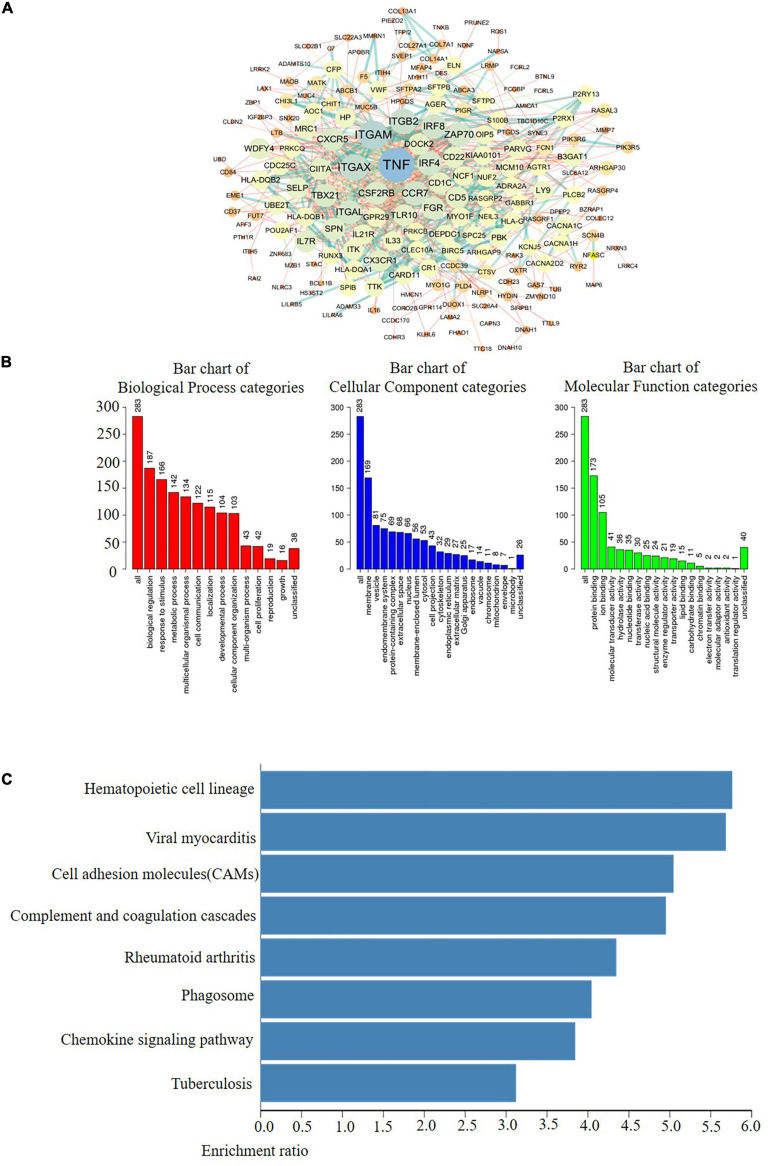
Coexpression network analysis of the AlkB family in LUAD. **(A)** The PPI network of AlkB family interaction partners generated by the frequently used c-BioPortal and Cytoscape. **(B)** Primary molecular functions, biological processes and cell components related to the biology of the AlkB family were identified using WebGestalt. **(C)** Bar plot of KEGG enriched terms analyzed by WebGestalt.

In fact, multiple of cellular proteins can work in a synergistic manner by forming multi-protein complexes. Moreover, protein-protein interactions (PPIs) play important roles among the different functions of a cell. Therefore, exploring PPI patterns is essential for understanding the structure and function of protein complexes (Planas-Iglesias et al., 2013; Furmanova et al., 2018). Afterward, to discover the PPI patterns between the differentially expressed AlkB family, we downloaded 248 co-expressed molecules with the highest correlation with AlkB family members using the cBioPortal database and modified them with Cytoscape (Figure 5A). These data suggest that TNF, ITGAM, ITGB2, ITGAX, DOCK2, IRF4, IRF8, CXCR5, CSF2RB, ZAP70, and TBX21 were primarily associated with the modulation and function of AlkB family in LUAD. In addition, GO analysis could be used to annotate the biological meaning of candidate biomarkers and their functions in various organisms. It is composed of molecular functions, biological processes and cellular components (Gene Ontology Consortium, 2019). And KEGG, a genome encyclopedia, can be used to perform biological interpretation of fully sequenced genomes and infer the systematic behaviors of targeted gene sets (Kanehisa et al., 2017). To probe the biological functions of these co-expressed molecules, WebGestalt was used to conduct GO annotation and KEGG pathway analysis for this study. GO annotation recognized that the AlkB family is mainly located on the cell membrane and is involved in biological regulation processes. In terms of molecular function, the family mainly participates in protein binding (Figure 5B). The corresponding pathways, as shown Figure 5C, were hematopoietic cell lineage, viral myocarditis and cell adhesion molecules (CAMs).

### Immune Cell Infiltration of the AlkB Family in LUAD Patients

**FIGURE 6:**
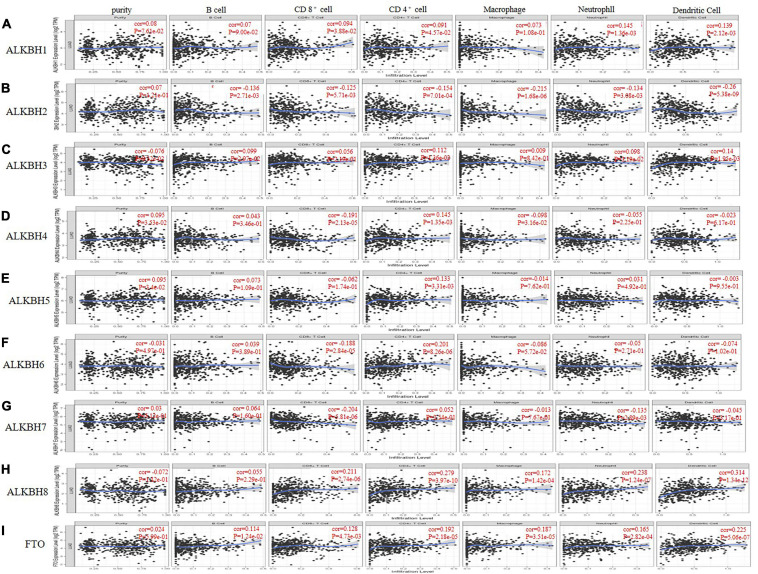
The correlations between differentially expressed AlkB family and immune cell infiltration. **(A–I)** The effect of ALKBH1-8 and FTO on the immune cell infiltration analyzed by TIMER2.0.

Members of the AlkB family are involved in the inflammatory response and the infiltration of immune cells in the tumor (Zhang et al., 2019a), thereby affecting the clinical outcomes of LUAD patients. The TIMER database was used to discuss the correlation between the differential expression of the AlkB family and immune cell infiltration. ALKBH1 and FTO were positively correlated with B cells, CD8 + T cells, CD4 + T cells, macrophages, neutrophils and dendritic cells (Figures 6A,I), but ALKBH2 was negatively correlated with these immune cells (Figure 6B). There were positive correlations between ALKBH3 expression and the infiltration of B cells, CD4 + cells, neutrophils and dendritic cells (Figure 6C) and negative correlations between ALKBH4 expression and the infiltration of CD8 + T cells and macrophages (Figure 6D). Similarly, ALKBH5 was positively correlated with CD4 + T cells (Figure 6E). In contrast, ALKBH6 and ALKBH7 were negatively correlated with CD8 + T cells and macrophages (Figures 6F,G). We also found that ALKBH8 was positively correlated with CD4 + T cells, CD8 + T cells, macrophages, neutrophils and dendritic cells (Figure 6H). We also evaluated the correlation between the expression levels of the AlkB family and immune cell infiltration. After adjusting for some confounding factors, including B cells, CD8 + T cells, CD4 + T cells, macrophages, neutrophils and dendritic cells, we used the Cox proportional hazard model to find that B cells were significantly associated with the clinical outcome of LUAD patients (Table 2).

**TABLE 2:** The Cox proportional hazard model of the ALKB family and six tumor-infiltrating immune cells in LUAD.

	**coef**	**HR**	**95%CI_l**	**95%CI_u**	***p* value**	**sig**
B cell	–5.066	0.006	0.000	0.088	0.000	***
CD8_Tcell	0.223	1.249	0.191	8.170	0.816	
CD4_Tcell	2.450	11.588	0.674	199.263	0.091	
Macrophage	–0.438	0.645	0.047	8.932	0.744	
Neutrophil	–0.368	0.692	0.014	33.222	0.852	
Dendritic	–0.038	0.962	0.249	3.714	0.955	
ALKBH1	–0.042	0.959	0.624	1.474	0.848	
ALKBH2	0.000	1.000	0.762	1.311	0.999	
ALKBH3	–0.088	0.915	0.720	1.164	0.471	
ALKBH4	–0.361	0.697	0.472	1.030	0.070	
ALKBH5	0.234	1.263	0.877	1.819	0.209	
ALKBH6	0.048	1.049	0.806	1.366	0.721	
ALKBH7	0.109	1.115	0.839	1.482	0.452	
ALKBH8	0.269	1.308	0.956	1.791	0.094	
FTO	–0.201	0.818	0.584	1.147	0.244	

***p < 0.001.

### Methylation Expression Levels of the AlkB Family in LUAD Patients

DNA methylation is negatively correlated with gene expression (Gensous et al., 2020; Kronfol et al., 2020). And we want to evaluate the effect of methylation on the expression levels of Alkb family members. We downloaded the methylation expression data of AlkB family members from the DiseaseMeth database. The results reflect that the methylation expression levels of ALKBH1, ALKBH2, and ALKBH6 are lower in LUAD samples than in normal samples. Furthermore, the methylation expression levels of ALKBH3 were higher in LUAD samples (Supplementary Figure 1). Previous findings indicated the upregulated ALKBH1/2 and downregulated ALKBH3 in LUAD samples (Figure 1). Thus, the abnormal expression of ALKBH1/2/3 might be due to the changes in their methylation values.

## Discussion

Emerging reports have revealed that DNA damage induces autophagy, apoptosis and senescence of cells, thus playing pivotal roles in the development of malignant tumors (Lord and Ashworth, 2012; Roos et al., 2016). As a classic reversal DNA repair enzyme, the AlkB family cannot be ignored in the development of multiple cancers (Xie et al., 2018; Zhu et al., 2019). At present, some scholars have discovered that ALKBH5 regulates epithelial-mesenchymal transition and angiogenesis to promote cancer progression (Panneerdoss et al., 2018). Chen et al. found that ALKBH3 can promote the proliferation, migration and invasion of cancer cells (Panneerdoss et al., 2018). Although a few members of the AlkB family have been discovered to play critical roles in lung cancer (Tasaki et al., 2011; Wu et al., 2011), the specific roles of other members of the AlkB family in LUAD have not yet been elucidated.

In this research, the AlkB family was studied comprehensively in terms of its expression and clinical prognostic value in LUAD. For the first time, we summarized the mRNA expression levels of nine members of the ALKBH family in LUAD compared with normal tissues. Next, a further novel finding is that the AlkB family is closely related to individual clinicopathological stages and lymph node metastasis. In addition, higher expression levels of ALKBH2/4/7 were significantly associated with shorter OS times. Lower expression levels of ALKBH1/3/8 and FTO were significantly associated with longer OS times. The patients with elevated ALKBH3/8 and FTO levels had long PPS, while patients with decreased ALKBH2 levels had shortened PPS. The process of tumor occurrence is intricate and multifaceted, and genetic alterations play a significant role in this process (Martincorena et al., 2017; Sondka et al., 2018; Coelho et al., 2019). The AlkB family shows evidence of frequent gene alterations in LUAD. The expression alteration of mRNA is one of the most typical mutations. These results comprehensively demonstrated that differential expression of the AlkB family probably plays a critical role in LUAD.

After discovering that the AlkB family is differentially expressed in LUAD and normal tissues, we next explored their molecular biological functions. We focused on the functional analysis of the AlkB family and their 247 frequently altered neighboring genes in LUAD. DNA repair- and protein methyltransferase activity-related genes, including ASCC3 and TRMT112, were highly linked to AlkB family alterations. Protein network interactions showed that TNF, ITGAM, ITGB2, ITGAX, DOCK2, IRF4, and IRF8 were primarily associated with the modulation and function of the differentially expressed AlkB family in LUAD. KEGG pathway analysis showed that AlkB family members were closely related to the CAMs. Multiple of CAM molecules have been proved to involve in the synaptic connection formation, maturation, function, and plasticity among cells. In a variety of human malignancies, including LUAD, tumor progression has been observed to be associated with altered CAM signaling pathways (Johnson, 1991). Similarly, Fujii and colleague reported the stimulative roles of ALKBH2 on the MUC1, an anti-adhesion molecule, on tumor cell growth (Fujii et al., 2013).

Increasing evidence shows that tumor progression and recurrence will be affected by immune cell infiltration and become an important determinant of immunotherapy response and clinical outcome (Lopes et al., 2019; Rotte, 2019). In our study, we found a significant correlation between the expression of the AlkB family and the infiltration of six immune cells, including B cells, CD8 + T cells, CD4 + T cells, macrophages, neutrophils and dendritic cells. Previous studies have shown that the relationship between tumor cells and immune microenvironment plays a key role in the occurrence and development of tumors (Varn et al., 2017). Many researchers have found that the infiltration of immune cells is closely related to the OS or disease-specific survival rate (DSS) of many cancers, including LUAD (Kadara et al., 2017; Hofman, 2020; Luo et al., 2020). As a kind of immune cells, B cells can fight against tumors through the production of antibodies, antigen royalty or the secretion of immune regulatory factors (Stoycheva et al., 2021). Han et al. (2020) found that B cells can be used as an independent indicator of OS prediction in LUAD. It is well known that methylation plays an important role in the development of cancer. In this study, we have found that Alkb family has abnormal expression of DNA methylation in LUAD. In fact, many researchers have also made some breakthroughs in the modification of RNA methylation in Alkb family. N6-methyladenosine is the most common RNA modification. ALKBH5, A member of the Alkb family, is the most widespread m^6^A demethylase. It has been shown that ALKBH5 can inhibit tumor growth and metastasis (Ji et al., 2020; Jin et al., 2020). Zhu et al. (2020) found that m^6^A-related gene ALKBH5 had a good predictive value in pathological staging and prognosis of LUAD. The results of our study reflect that the AlkB family not only can be used as prognostic indicators but also reflect immune status.

In this study, we comprehensively analyzed the expression and prognosis of the AlkB family in LUAD from the perspective of bioinformatics. Through a comprehensive analysis, we found that high ALKBH2 expression was significantly related to poor OS and PPS in LUAD patients. In addition, abnormally expressed ALKBH2 could also alter the immune cell infiltration in LUAD. These results provide novel biomarkers for the diagnosis of LUAD in the future and will help clinicians design potential therapeutic strategies for LUAD patients to improve the survival rate.

## Data Availability Statement

The original contributions presented in the study are included in the article/Supplementary Material, further inquiries can be directed to the corresponding authors.

## Author Contributions

GW, JL, and ZX: acquisition of the data. YY: analysis and interpretation of the data. JZ: conception and design. YC and BP: data curation. JH: development of the methodology. GW and ZX: writing the manuscript. All authors contributed to the article and approved the submitted version.

## Conflict of Interest

The authors declare that the research was conducted in the absence of any commercial or financial relationships that could be construed as a potential conflict of interest.

## Footnotes

1 http://www.oncomine.org

2 http://gepia.cancer-pku.cn/index.html

3 http://ualcan.path.uab.edu/index.html

4 http://kmplot.com/analysis/

5 http://cbioportal.org

6 http://genemania.org

7 http://www.webgestalt.org

8 https://cistrome.shinyapps.io/timer/

9 http://bioinfo.hrbmu.edu.cn/diseasemeth/

## Supplementary Material

Supplementary Figure 1 The methylation expression of AlkB family members in LUAD patients (DiseaseMeth).
