# Supplementary figures and images for: ALKBH1-8 and FTO: Potential Therapeutic Targets and Prognostic Biomarkers in Lung Adenocarcinoma Pathogenesis
Source: Front Cell Dev Biol. 2021 Jun 3;9:633927. doi: 10.3389/fcell.2021.633927 (PMC8209387; doi:10.3389/fcell.2021.633927)

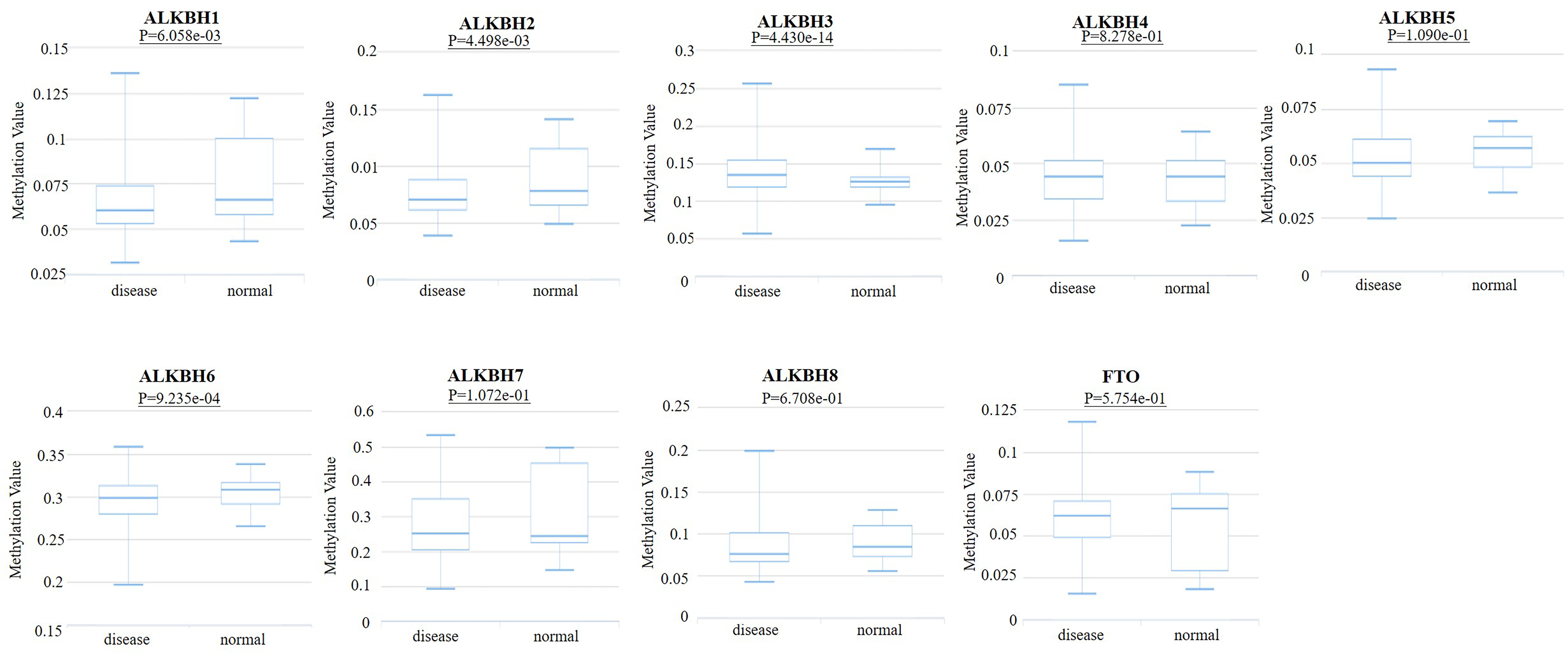

Supplement: Supplementary Figure 1 — The methylation expression of AlkB family members in LUAD patients (DiseaseMeth). [file Image_1.TIF]
