# Supplementary material for: ALKBH1-8 and FTO: Potential Therapeutic Targets and Prognostic Biomarkers in Lung Adenocarcinoma Pathogenesis
Source: Front Cell Dev Biol. 2021 Jun 3;9:633927. doi: 10.3389/fcell.2021.633927 (PMC8209387; doi:10.3389/fcell.2021.633927)
Supplement: Supplementary file 3 [file Table_2.docx]

**Table S2:** The Alkb family member-associated co-expressed molecules in LUAD.

| Gene | Log Ratio | p-Value | expression |
| --- | --- | --- | --- |
| PALD1 | -0.76 | 6.04E-09 | Unaltered group |
| RAI2 | -0.8 | 1.47E-08 | Unaltered group |
| TMEM273 | -0.84 | 2.65E-08 | Unaltered group |
| WDFY4 | -0.96 | 3.63E-08 | Unaltered group |
| ARHGAP30 | -0.8 | 3.95E-08 | Unaltered group |
| PIK3R6 | -0.96 | 5.25E-08 | Unaltered group |
| VWF | -0.88 | 5.92E-08 | Unaltered group |
| RASGRP2 | -1.16 | 9.35E-08 | Unaltered group |
| ITIH5 | -1.23 | 1.12E-07 | Unaltered group |
| LAMA2 | -0.82 | 1.15E-07 | Unaltered group |
| COL27A1 | -1.09 | 1.61E-07 | Unaltered group |
| ZNF521 | -0.81 | 1.73E-07 | Unaltered group |
| DNAH1 | -1 | 2.22E-07 | Unaltered group |
| CCR6 | -0.99 | 2.30E-07 | Unaltered group |
| CSF2RB | -0.87 | 2.59E-07 | Unaltered group |
| GAS7 | -0.78 | 2.59E-07 | Unaltered group |
| ZNF154 | -0.82 | 2.93E-07 | Unaltered group |
| CBFA2T3 | -0.85 | 3.68E-07 | Unaltered group |
| CASS4 | -0.77 | 4.64E-07 | Unaltered group |
| TBX4 | -0.83 | 4.91E-07 | Unaltered group |
| FMO2 | -0.95 | 5.10E-07 | Unaltered group |
| CIITA | -0.88 | 5.11E-07 | Unaltered group |
| NAPSB | -0.84 | 5.32E-07 | Unaltered group |
| LSAMP | -0.87 | 6.28E-07 | Unaltered group |
| APOBR | -0.85 | 6.91E-07 | Unaltered group |
| CCDC146 | -1.01 | 7.55E-07 | Unaltered group |
| RASAL3 | -0.81 | 7.75E-07 | Unaltered group |
| PRUNE2 | -0.96 | 1.11E-06 | Unaltered group |
| IL16 | -0.77 | 1.18E-06 | Unaltered group |
| PLCB2 | -0.75 | 1.19E-06 | Unaltered group |
| SPN | -0.91 | 1.29E-06 | Unaltered group |
| CDH23 | -1.06 | 1.30E-06 | Unaltered group |
| BOC | -0.77 | 1.31E-06 | Unaltered group |
| NLRP1 | -0.82 | 1.33E-06 | Unaltered group |
| CORO2B | -0.86 | 1.38E-06 | Unaltered group |
| GABBR1 | -0.89 | 1.41E-06 | Unaltered group |
| COL13A1 | -0.99 | 1.51E-06 | Unaltered group |
| PLXNC1 | -0.8 | 1.53E-06 | Unaltered group |
| ANOS1 | -0.85 | 1.67E-06 | Unaltered group |
| MYO1F | -0.75 | 1.96E-06 | Unaltered group |
| ABI3BP | -1.02 | 2.08E-06 | Unaltered group |
| LINC00341 | -0.85 | 2.10E-06 | Unaltered group |
| PDZRN3 | -0.78 | 2.11E-06 | Unaltered group |
| AGAP2 | -0.76 | 2.18E-06 | Unaltered group |
| CD22 | -1.23 | 2.22E-06 | Unaltered group |
| CARD11 | -0.79 | 2.26E-06 | Unaltered group |
| ABCA9 | -0.92 | 2.40E-06 | Unaltered group |
| FGD2 | -0.81 | 2.78E-06 | Unaltered group |
| CHIT1 | -1.83 | 2.94E-06 | Unaltered group |
| PKNOX2 | -1.04 | 3.04E-06 | Unaltered group |
| PCDHGB7 | -0.83 | 3.25E-06 | Unaltered group |
| LOC728392 | -0.81 | 3.30E-06 | Unaltered group |
| HMCN1 | -0.88 | 3.86E-06 | Unaltered group |
| C7 | -1.3 | 4.25E-06 | Unaltered group |
| SNX20 | -0.79 | 4.39E-06 | Unaltered group |
| NLRC3 | -0.78 | 5.63E-06 | Unaltered group |
| CACNA1C | -0.83 | 5.69E-06 | Unaltered group |
| MAP6 | -0.84 | 5.86E-06 | Unaltered group |
| TOX2 | -0.75 | 6.25E-06 | Unaltered group |
| PARP15 | -1.13 | 6.42E-06 | Unaltered group |
| LTB | -0.94 | 6.65E-06 | Unaltered group |
| SUGT1P4-STRA6LP-CCDC180 | -1.01 | 6.73E-06 | Unaltered group |
| TNFAIP2 | -0.79 | 6.77E-06 | Unaltered group |
| ACAP1 | -0.85 | 7.07E-06 | Unaltered group |
| CD37 | -0.84 | 7.36E-06 | Unaltered group |
| DOCK2 | -0.81 | 7.41E-06 | Unaltered group |
| MFAP4 | -0.99 | 7.60E-06 | Unaltered group |
| KLHL6 | -0.92 | 7.84E-06 | Unaltered group |
| UBE2T | 0.78 | 7.91E-06 | Altered group |
| TNFRSF14-AS1 | -0.81 | 7.98E-06 | Unaltered group |
| CR1 | -1.03 | 7.98E-06 | Unaltered group |
| NRXN3 | -0.95 | 8.00E-06 | Unaltered group |
| SLCO2B1 | -0.77 | 8.62E-06 | Unaltered group |
| PTGDS | -0.99 | 9.36E-06 | Unaltered group |
| SULT1C4 | -0.81 | 9.38E-06 | Unaltered group |
| RTN1 | -0.8 | 1.01E-05 | Unaltered group |
| IRF8 | -0.79 | 1.04E-05 | Unaltered group |
| PIEZO2 | -0.83 | 1.04E-05 | Unaltered group |
| RUNX3 | -0.76 | 1.07E-05 | Unaltered group |
| JAML | -0.83 | 1.11E-05 | Unaltered group |
| PIK3R5 | -0.79 | 1.17E-05 | Unaltered group |
| CCR7 | -0.93 | 1.26E-05 | Unaltered group |
| RASGRP4 | -0.77 | 1.29E-05 | Unaltered group |
| ADGRG2 | -0.98 | 1.31E-05 | Unaltered group |
| ARHGAP9 | -0.75 | 1.32E-05 | Unaltered group |
| SDK2 | -1.16 | 1.37E-05 | Unaltered group |
| IRAK3 | -0.76 | 1.43E-05 | Unaltered group |
| ABCB1 | -0.86 | 1.50E-05 | Unaltered group |
| PRAM1 | -0.88 | 1.51E-05 | Unaltered group |
| SLIT3 | -1.01 | 1.60E-05 | Unaltered group |
| PRKCB | -0.76 | 1.61E-05 | Unaltered group |
| ITGAL | -0.78 | 1.63E-05 | Unaltered group |
| BCL11B | -0.79 | 1.97E-05 | Unaltered group |
| HCG26 | -0.77 | 1.99E-05 | Unaltered group |
| TBC1D10C | -0.82 | 2.24E-05 | Unaltered group |
| ITGB2 | -0.78 | 2.38E-05 | Unaltered group |
| PLD4 | -0.91 | 2.44E-05 | Unaltered group |
| ADAMTS10 | -0.79 | 2.50E-05 | Unaltered group |
| ABCA6 | -0.77 | 2.51E-05 | Unaltered group |
| IL21R | -0.76 | 2.53E-05 | Unaltered group |
| OIP5 | 0.76 | 2.59E-05 | Altered group |
| PARVG | -0.77 | 2.72E-05 | Unaltered group |
| MATK | -0.82 | 2.82E-05 | Unaltered group |
| CCDC39 | -1.08 | 3.07E-05 | Unaltered group |
| SELP | -0.94 | 3.17E-05 | Unaltered group |
| ITGAM | -0.81 | 3.57E-05 | Unaltered group |
| EME1 | 0.76 | 3.67E-05 | Altered group |
| ZAP70 | -0.91 | 3.73E-05 | Unaltered group |
| SPC25 | 0.77 | 3.78E-05 | Altered group |
| PDZD4 | -0.82 | 3.85E-05 | Unaltered group |
| GVINP1 | -0.84 | 3.89E-05 | Unaltered group |
| ZNF662 | -0.76 | 3.90E-05 | Unaltered group |
| ITIH4 | -0.89 | 3.90E-05 | Unaltered group |
| TNXB | -1.03 | 3.94E-05 | Unaltered group |
| RYR2 | -0.89 | 4.25E-05 | Unaltered group |
| PLPPR4 | -0.78 | 4.27E-05 | Unaltered group |
| PCLAF | 0.78 | 4.38E-05 | Altered group |
| GOLGA8A | -0.82 | 4.56E-05 | Unaltered group |
| NHLRC4 | -0.9 | 4.57E-05 | Unaltered group |
| SEC31B | -0.87 | 4.74E-05 | Unaltered group |
| CTSV | 1.06 | 4.81E-05 | Altered group |
| TTK | 0.9 | 4.90E-05 | Altered group |
| ZNF831 | -0.95 | 5.04E-05 | Unaltered group |
| TSPOAP1 | -0.78 | 5.40E-05 | Unaltered group |
| CFP | -0.82 | 5.58E-05 | Unaltered group |
| PCDHGB6 | -0.91 | 5.62E-05 | Unaltered group |
| P2RX1 | -0.82 | 5.71E-05 | Unaltered group |
| MAOB | -0.77 | 5.77E-05 | Unaltered group |
| P2RY13 | -0.78 | 6.04E-05 | Unaltered group |
| TUB | -0.75 | 6.38E-05 | Unaltered group |
| RUBCNL | -0.76 | 6.81E-05 | Unaltered group |
| LRRC4 | -0.89 | 6.87E-05 | Unaltered group |
| GPR162 | -0.86 | 7.00E-05 | Unaltered group |
| NFASC | -0.86 | 7.24E-05 | Unaltered group |
| CTTNBP2 | -0.84 | 8.74E-05 | Unaltered group |
| C16ORF54 | -0.76 | 9.00E-05 | Unaltered group |
| HYDIN | -1.26 | 9.19E-05 | Unaltered group |
| ITGAX | -0.79 | 9.20E-05 | Unaltered group |
| NKD2 | -0.83 | 9.28E-05 | Unaltered group |
| TLR10 | -0.95 | 9.57E-05 | Unaltered group |
| SVEP1 | -0.8 | 9.62E-05 | Unaltered group |
| CAPN3 | -0.87 | 9.66E-05 | Unaltered group |
| SPATA18 | -1.17 | 9.74E-05 | Unaltered group |
| ADAM33 | -1.07 | 1.01E-04 | Unaltered group |
| EPHB6 | -1.19 | 1.02E-04 | Unaltered group |
| CPNE5 | -0.84 | 1.03E-04 | Unaltered group |
| MEIOC | 0.76 | 1.04E-04 | Altered group |
| CELF6 | -0.79 | 1.06E-04 | Unaltered group |
| PPP1R16B | -0.79 | 1.07E-04 | Unaltered group |
| IL33 | -0.81 | 1.13E-04 | Unaltered group |
| CLEC10A | -0.82 | 1.15E-04 | Unaltered group |
| BIRC5 | 0.96 | 1.20E-04 | Altered group |
| LY9 | -0.76 | 1.24E-04 | Unaltered group |
| FGR | -0.75 | 1.29E-04 | Unaltered group |
| PLCH2 | -0.96 | 1.37E-04 | Unaltered group |
| SIRPB1 | -0.77 | 1.37E-04 | Unaltered group |
| HS3ST2 | -1.08 | 1.38E-04 | Unaltered group |
| LINC00926 | -0.99 | 1.40E-04 | Unaltered group |
| HLA-DQA1 | -0.98 | 1.41E-04 | Unaltered group |
| DPEP2 | -0.78 | 1.41E-04 | Unaltered group |
| ADGRG5 | -0.87 | 1.45E-04 | Unaltered group |
| MUC5B | -1.95 | 1.58E-04 | Unaltered group |
| CARMN | -0.76 | 1.64E-04 | Unaltered group |
| SLC6A12 | -0.79 | 1.71E-04 | Unaltered group |
| CD5 | -0.8 | 1.72E-04 | Unaltered group |
| TNF | -0.87 | 1.75E-04 | Unaltered group |
| CXCR5 | -1.09 | 1.81E-04 | Unaltered group |
| NUF2 | 0.85 | 1.95E-04 | Altered group |
| COLEC12 | -0.84 | 2.03E-04 | Unaltered group |
| CDC25C | 0.91 | 2.05E-04 | Altered group |
| FCN1 | -0.77 | 2.10E-04 | Unaltered group |
| NCF1 | -0.77 | 2.10E-04 | Unaltered group |
| MIR4697HG | -1.06 | 2.13E-04 | Unaltered group |
| FCMR | -0.77 | 2.29E-04 | Unaltered group |
| MMRN1 | -0.86 | 2.47E-04 | Unaltered group |
| COL14A1 | -0.81 | 2.53E-04 | Unaltered group |
| AFF3 | -0.83 | 2.64E-04 | Unaltered group |
| PRKCQ | -0.79 | 2.64E-04 | Unaltered group |
| HLA-DQB1 | -0.8 | 2.65E-04 | Unaltered group |
| CD84 | -0.77 | 2.75E-04 | Unaltered group |
| MYO1G | -0.75 | 2.81E-04 | Unaltered group |
| SCN4B | -0.82 | 2.86E-04 | Unaltered group |
| MYH11 | -0.96 | 2.93E-04 | Unaltered group |
| C1QTNF7 | -0.89 | 3.07E-04 | Unaltered group |
| ABCA3 | -0.94 | 3.16E-04 | Unaltered group |
| ATP6V0D2 | -1.1 | 3.51E-04 | Unaltered group |
| MIAT | -0.95 | 3.76E-04 | Unaltered group |
| IL7R | -0.75 | 4.02E-04 | Unaltered group |
| FUT7 | -0.77 | 4.34E-04 | Unaltered group |
| CCDC170 | -1.04 | 4.34E-04 | Unaltered group |
| CHI3L2 | -0.93 | 4.54E-04 | Unaltered group |
| CX3CR1 | -0.8 | 4.57E-04 | Unaltered group |
| ROS1 | -1.09 | 5.03E-04 | Unaltered group |
| ADRA2A | -0.9 | 5.12E-04 | Unaltered group |
| CFAP70 | -0.95 | 5.19E-04 | Unaltered group |
| LILRB5 | -0.86 | 5.29E-04 | Unaltered group |
| NDNF | -0.78 | 5.52E-04 | Unaltered group |
| SPEG | -0.86 | 5.76E-04 | Unaltered group |
| TBX21 | -0.75 | 6.01E-04 | Unaltered group |
| ITK | -0.75 | 6.23E-04 | Unaltered group |
| NCF1C | -0.84 | 6.59E-04 | Unaltered group |
| DRC3 | -1.04 | 6.63E-04 | Unaltered group |
| PBK | 0.78 | 6.65E-04 | Altered group |
| MRC1 | -0.82 | 6.71E-04 | Unaltered group |
| DUOX1 | -0.99 | 7.48E-04 | Unaltered group |
| LRMP | -0.76 | 7.78E-04 | Unaltered group |
| MUC4 | -1.05 | 8.01E-04 | Unaltered group |
| MCM10 | 0.78 | 8.12E-04 | Altered group |
| FCGBP | -1.11 | 8.49E-04 | Unaltered group |
| ELN | -0.82 | 8.72E-04 | Unaltered group |
| BTNL9 | -0.81 | 8.90E-04 | Unaltered group |
| CD1C | -0.95 | 9.05E-04 | Unaltered group |
| LILRA6 | -0.78 | 9.05E-04 | Unaltered group |
| TCTEX1D1 | -0.91 | 9.44E-04 | Unaltered group |
| TTLL9 | -0.91 | 9.99E-04 | Unaltered group |
| DEPDC1 | 0.78 | 1.02E-03 | Altered group |
| DES | -1 | 1.09E-03 | Unaltered group |
| VILL | -0.92 | 1.14E-03 | Unaltered group |
| LAX1 | -0.79 | 1.14E-03 | Unaltered group |
| ADAM6 | -0.93 | 1.15E-03 | Unaltered group |
| MGAT3 | -0.87 | 1.17E-03 | Unaltered group |
| ZBP1 | -0.75 | 1.17E-03 | Unaltered group |
| SPIB | -1.03 | 1.19E-03 | Unaltered group |
| IRF4 | -0.82 | 1.30E-03 | Unaltered group |
| CDHR3 | -1.43 | 1.31E-03 | Unaltered group |
| CPAMD8 | -0.93 | 1.33E-03 | Unaltered group |
| LRRN4 | -1.04 | 1.37E-03 | Unaltered group |
| CACNA1H | -0.9 | 1.38E-03 | Unaltered group |
| TPSB2 | -0.94 | 1.46E-03 | Unaltered group |
| KCNJ5 | -0.84 | 1.56E-03 | Unaltered group |
| TPPP3 | -0.94 | 1.56E-03 | Unaltered group |
| CHI3L1 | -0.79 | 1.58E-03 | Unaltered group |
| HP | -1.45 | 1.73E-03 | Unaltered group |
| NAPSA | -0.77 | 2.03E-03 | Unaltered group |
| SLC46A2 | -0.9 | 2.05E-03 | Unaltered group |
| COL7A1 | -1.08 | 2.11E-03 | Unaltered group |
| PTH1R | -0.75 | 2.17E-03 | Unaltered group |
| SLC22A3 | -0.82 | 2.36E-03 | Unaltered group |
| FAM107A | -0.79 | 2.53E-03 | Unaltered group |
| CDH6 | -0.75 | 2.54E-03 | Unaltered group |
| PKDCC | -0.76 | 2.55E-03 | Unaltered group |
| HLA-DQB2 | -0.84 | 2.62E-03 | Unaltered group |
| MMP7 | -1.03 | 2.67E-03 | Unaltered group |
| PCDHGA10 | -0.79 | 3.03E-03 | Unaltered group |
| FHAD1 | -0.9 | 3.14E-03 | Unaltered group |
| POU2AF1 | -0.86 | 3.24E-03 | Unaltered group |
| RTN4RL1 | -0.94 | 3.34E-03 | Unaltered group |
| AGTR1 | -0.75 | 3.37E-03 | Unaltered group |
| ZMYND10 | -1.07 | 3.50E-03 | Unaltered group |
| CACNA2D2 | -1.02 | 3.55E-03 | Unaltered group |
| UBD | -0.82 | 3.91E-03 | Unaltered group |
| IGF2BP3 | 1.01 | 4.30E-03 | Altered group |
| ZNF683 | -0.79 | 4.39E-03 | Unaltered group |
| STAC | -0.77 | 4.42E-03 | Unaltered group |
| TESMIN | 0.76 | 4.49E-03 | Altered group |
| LGR6 | -0.78 | 4.53E-03 | Unaltered group |
| C5ORF49 | -0.94 | 4.96E-03 | Unaltered group |
| DNAH10 | -0.96 | 5.04E-03 | Unaltered group |
| HPGDS | -0.84 | 5.72E-03 | Unaltered group |
| SFTPB | -0.95 | 6.33E-03 | Unaltered group |
| OXTR | -0.75 | 6.59E-03 | Unaltered group |
| S100B | -0.8 | 6.64E-03 | Unaltered group |
| SFTPA2 | -1.24 | 6.65E-03 | Unaltered group |
| SFTPD | -1.03 | 6.91E-03 | Unaltered group |
| MZB1 | -0.75 | 7.07E-03 | Unaltered group |
| ECT2L | -1.08 | 7.22E-03 | Unaltered group |
| NEIL3 | 0.75 | 7.49E-03 | Altered group |
| ANKRD36BP2 | -0.88 | 8.89E-03 | Unaltered group |
| VSIG1 | -1.21 | 9.32E-03 | Unaltered group |
| HLA-G | -0.76 | 9.39E-03 | Unaltered group |
| FCRL5 | -0.8 | 9.80E-03 | Unaltered group |
| F5 | -0.98 | 0.0105 | Unaltered group |
| LRRK2 | -0.75 | 0.0106 | Unaltered group |
| KCNRG | -0.76 | 0.0115 | Unaltered group |
| B3GAT1 | -0.88 | 0.0117 | Unaltered group |
| CLDN2 | -1.41 | 0.0118 | Unaltered group |
| AOC1 | -0.96 | 0.0123 | Unaltered group |
| RASGRF1 | -0.82 | 0.0127 | Unaltered group |
| FCRL2 | -0.79 | 0.0143 | Unaltered group |
| PRG4 | -0.84 | 0.015 | Unaltered group |
| CRLF1 | -1.02 | 0.0167 | Unaltered group |
| AGER | -0.84 | 0.0247 | Unaltered group |
| PIGR | -0.89 | 0.0254 | Unaltered group |
| TMEM59L | -0.89 | 0.0332 | Unaltered group |
| TFPI2 | 0.84 | 0.0377 | Altered group |
| SLC26A4 | -0.88 | 0.0428 | Unaltered group |
